# Histological assessment of a novel restorative coronary artery bypass graft in a chronic ovine model

**DOI:** 10.3389/fbioe.2025.1488794

**Published:** 2025-02-10

**Authors:** Yu Sato, Matthew Kutnya, Biniyam Abebe, Mohammed S. El Kurdi, Martijn Cox, Richard W. Bianco, Bart Meuris, Yoshinobu Onuma, Patrick W. Serruys, Renu Virmani

**Affiliations:** ^1^ CVPath Institute, Gaithersburg, MD, United States; ^2^ Xeltis Eindhoven, Eindhoven, Netherlands; ^3^ Experimental Surgical Services, University of Minneapolis, Minneapolis, MN, United States; ^4^ Department of Cardiac Surgery, University Hospital Leuven, Leuven, Belgium; ^5^ Department of Cardiology, National University of Ireland, Galway (NUIG), Galway, Ireland

**Keywords:** coronary artery disease, coronary artery bypass grafting, saphenous vein graft, small diameter vascular graft, tissue-engineered vascular graft, large animal model, ovine model

## Abstract

**Background:**

Although prosthetic conduits for coronary artery bypass grafting (CABG) are increasingly needed because of the limited availability and patency of autologous conduits, no alternatives have succeeded.

**Methods:**

Sixteen sheep underwent CABG. Thirteen received a bioabsorbable polymer graft with an incorporated nitinol microskeleton (Xeltis coronary artery bypass graft [XABG]), and three received autologous saphenous vein grafts (SVG). Pathological evaluation was conducted at 12 months.

**Results:**

In the XABG group, two sheep died perioperatively; two were sacrificed at 3 months (1 occluded, 1 patent) and two at 6 months (both patent). Two more died from occlusion at 9–10 months, and five survived with patent grafts at 12 months. All SVGs remained patent for 12 months. Histology demonstrated near-complete luminal endothelialization in XABG, with increased polymer adsorption and matrix deposition. The cross-sectional area of the SVG lumen was significantly larger than XABGs (48.2 mm^2^ vs 12.9 mm^2^, p = 0.0018), consistent with a reduced angiographic flow velocity in SVG. The neointimal area was greater in SVGs than XABGs (19.6 vs. 6.7 mm^2^, p = 0.0005), especially at the distal ends of SVGs due to thrombus formation.

**Conclusion:**

XABG demonstrated 1-year feasibility with consistent endothelialization and polymer absorption. While SVGs had better patency, they showed greater diametrical irregularity and subsequent neointimal proliferation.

## Introduction

Autologous saphenous vein grafts (SVGs) and internal mammary arteries (IMAs) are commonly used for bypassing stenotic coronary arteries. It is well known that the SVG failure rates vary from 10% to 30% at 1 year and reach approximately 50% at 5–10 years, whereas the patency rate for IMA is reported to be approximately 90%–95% at 10–15 years (Alexander et al., 2005; Taggart, 2013).

An off-the-shelf, synthetic, and small-diameter vascular graft (SDVG) that can remain patent in the challenging CABG circulation could create a worldwide paradigm shift in the treatment of millions of patients annually. Although there are clinical reports of synthetic ePTFE grafts being used with some success in CABG (Sapsford et al., 1981; Laube et al., 2000; Dohmen et al., 2013), including some clinical trials performed (Emery et al., 1996; Weyand et al., 1999), there are currently no approved synthetic grafts clinically indicated for CABG use.

**FIGURE 1:**
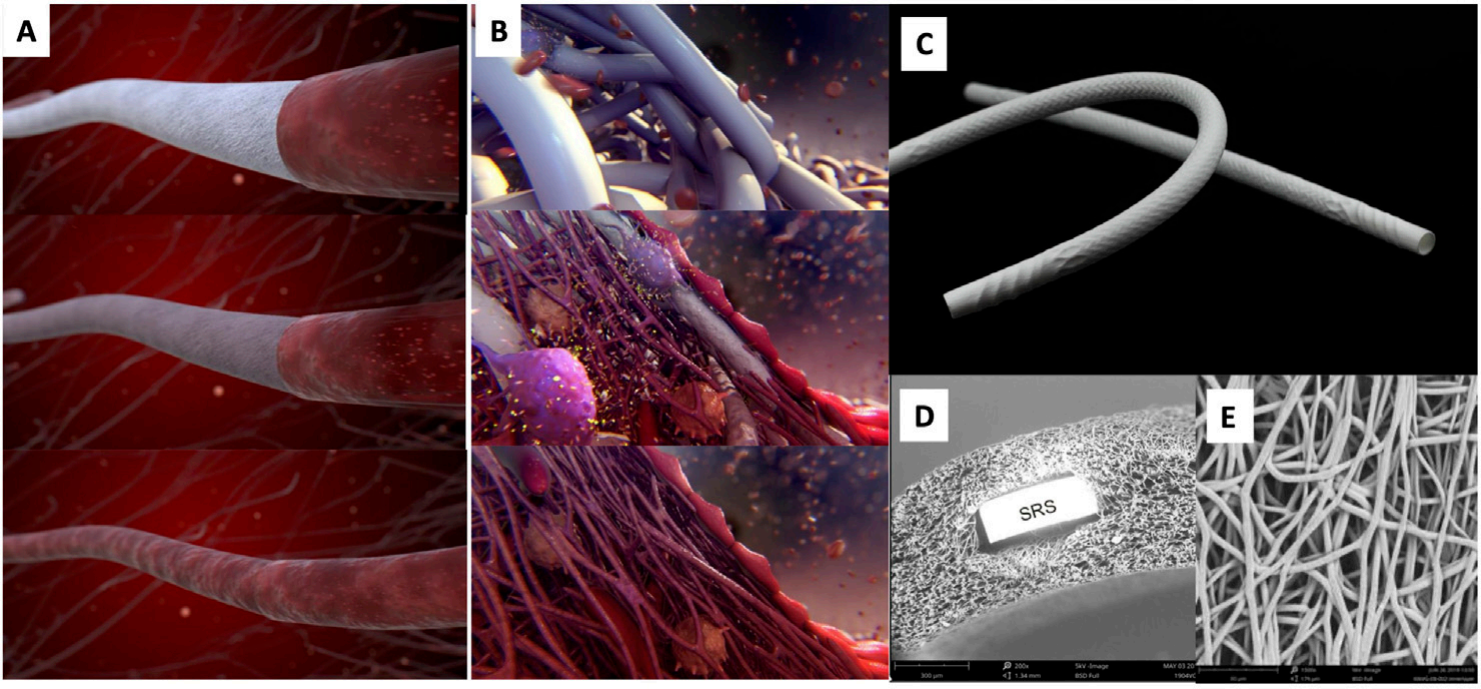
Artistic visualization of the ETR process of the device **(A)** and at the cellular level **(B)**. An XABG photo is shown in **(C)**. SEM cross-section **(D)** shows the device microstructure with one strut of the built-in nitinol SRS. SEM luminal view **(E)** shows the microporosity of the device. The polymer is manufactured into a microporous graft using electrospinning and incorporates a nitinol microskeleton (strain relief system or SRS) to enhance kink resistance. Modified and reproduced from Ono et al. (2023).

Recently, a new technology has been proposed using a bioabsorbable polymer matrix that allows endogenous tissue repair (ETR) without the use of stem cells or animal-derived products (Mes et al., 2022). The prosthesis is infiltrated by the patient’s own cells, triggering a series of physiological events that lead to ETR, gradually replacing the native tissue. We developed a 15-cm-long, 4-mm inner diameter restorative bypass graft composed of a bioabsorbable polymer based on the supramolecular ureidopyrimidinone motif (Webber et al., 2015; Sijbesma et al., 1997) (Figure 1). This class of polymers is currently under clinical investigation for various cardiovascular applications (Mes et al., 2022).

The purpose of the study was to evaluate the feasibility and long-term patency of implanted restorative SDVGs. We observed the evolution of lumen geometry via serial angiographic assessment and studied the histologic changes of the novel restorative SDVG compared to an autologous SVG control in an ovine CABG model.

## Materials and methods

### Animal study design

The study was conducted in accordance with the Guide for Care and Use of Laboratory Animals and the ARRIVE guidelines (Animal Research: Reporting of *In Vivo* Experiments). The study protocol was approved by the Institutional Animal Care and Use Committee (IACUC) and the Ethical Committee of the American Preclinical Services (Minneapolis, MN, United States) (Protocols IQI001-IS02, IQI005-IS02) before study initiation and performed in regulation and compliance to guidelines.

Sixteen Suffolk sheep with a body weight of approximately 60 kg were used in this study: thirteen were implanted with a restorative Xeltis coronary artery bypass graft (XABG), and three were implanted with an SVG. Only one graft was implanted per animal to connect the descending aorta to the LAD to bypass the proximal LAD, as previously described (Shofti et al., 2004). All animals were given aspirin and clopidogrel. Implantation, angiography, and termination were performed under general anesthesia using 2–8 mg/kg propofol and 0%–5% isoflurane. The restorative SDVGs or autologous SVGs were implanted under cardiopulmonary bypass, followed by ligation of the LAD at a few millimeters upstream of the graft distal anastomosis.

Animals were initially intended to be terminated after 6-month survival, and the safety of the restorative SDVG was to be compared to the SVG control. However, at 6 months, there were few complications, and angiography showed promising results. Therefore, the study was extended to 12 months. Humane euthanasia was ensured by intravenous administration of an appropriate barbiturate per animal study site protocol.

### Angiography imaging and analysis

Serial angiography was performed at the time of implant and 1-month, 3-month, 6-month, 9-month (XABG only), and 12-month follow-up. Quantitative coronary angiography with edge detection and videodensitometry, volumetric flow velocity, and optical coherence tomography (OCT) was performed at 3 months, 6 months, 9 months, and 12 months according to standard procedures described elsewhere (Wu et al., 2022) and was analyzed by an independent core laboratory (CORRIB Corelab, National University of Ireland Galway, Galway, Ireland).

### Histopathology and *ex vivo* analyses

#### Gross/radiographic/microCT examination process

The whole heart and attached aorta with a graft were perfusion-fixed with 10% buffered formalin under physiological pressure following the termination of a heparinized animal. The specimen was sent to CVPath Institute for further examination. Upon receipt of the heart, the pericardium was removed, and a radiograph and microCT were performed. Then, the graft with both the proximal and distal anastomosis sites intact was removed from the heart, radiographed, and stained by iodine for microCT image acquisition (details are in Supplementary Methods).

#### Histopathological examination

The proximal anastomosis was separated for longitudinal cutting through the ostium of the anastomosis, and the middle-graft cross-sections [n = 4 (2 cm each), proximal, distal, and two sections from the middle] were submitted for Spurr embedding and stained with hematoxylin and eosin (H&E). Segments of the distal anastomosis and its distal sections were processed in ethanol and xylene, embedded in paraffin, sectioned at 4–6 mm, and stained with H&E and Movat pentachrome. Histologic evaluation of XABG was performed to semi-quantitatively assess the extent of polymer absorption, matrix deposition, inflammation, angiogenesis, and neointima formation in all animals (Supplementary Table S1). In selected XABG sections, immunohistochemical staining, transmission electron microscopy (TEM), and scanning electron microscopy (SEM) analyses were performed to evaluate the luminal surface endothelial lining. Details are in Supplementary Methods.

#### Morphometric analysis of microCT cross-sections and histologic sections in 12-month animals

In the 12-month follow-up of animals with XABG and SVG, morphometrical analyses of both microCT and histologic cross-sections were conducted using computerized planimetry (Zen2, blue edition, Carl Zeiss, Oberkochen, Germany). For microCT analysis, graft cross-section images were captured every 10 mm from the proximal to distal segments, and the luminal and intima-graft border areas were measured. In histomorphometry analysis, digitized histology slides were utilized to measure luminal and intima-media border areas. Neointimal area and percent area stenosis were then calculated accordingly (Supplementary Figure S1). Ostial sections were excluded from the analysis due to their longitudinal cut and inability to measure these features. Details are shown in Supplementary Methods.

### Statistics

The Student’s t-test was used to analyze the significance of differences for continuous variables with normal distributions, whereas comparisons of variables with non-parametric distribution were assessed by the Kruskal–Wallis test. All analyses were performed using JMP software (version 15.0, SAS Institute, Inc., NC, United States). A p-value of <0.05 was considered statistically significant.

## Results

### Animal study

Supplementary Figure S2 summarizes the follow-up of Suffolk sheep (n = 16) with either XABG (N = 13) or SVG control (N = 3). Two animals with XABG did not survive the perioperative phase. Two animals with XABG were electively sacrificed after the 3-month angiographic assessment. One graft was occluded, and the other revealed distal stenosis and was sacrificed for exploratory learning (graft patent). Subsequently, two more animals with XABG were electively sacrificed as a result of the 6-month angiography findings. One graft showed focal aneurysmal change, and another showed dissection with neointimal perforation due to OCT catheterization, and both were patent at sacrifice. More details were described previously (Ono et al., 2023). The remaining seven animals with XABG survived until the exploratory 12-month time point; however, two unscheduled deaths occurred at 8.5 months and 9.5 months due to acute myocardial infarction. Finally, five animals survived with patent XABGs until the 12-month follow-up. All three SVG controls were patent at 12 months.

### Angiographic findings

**FIGURE 2:**
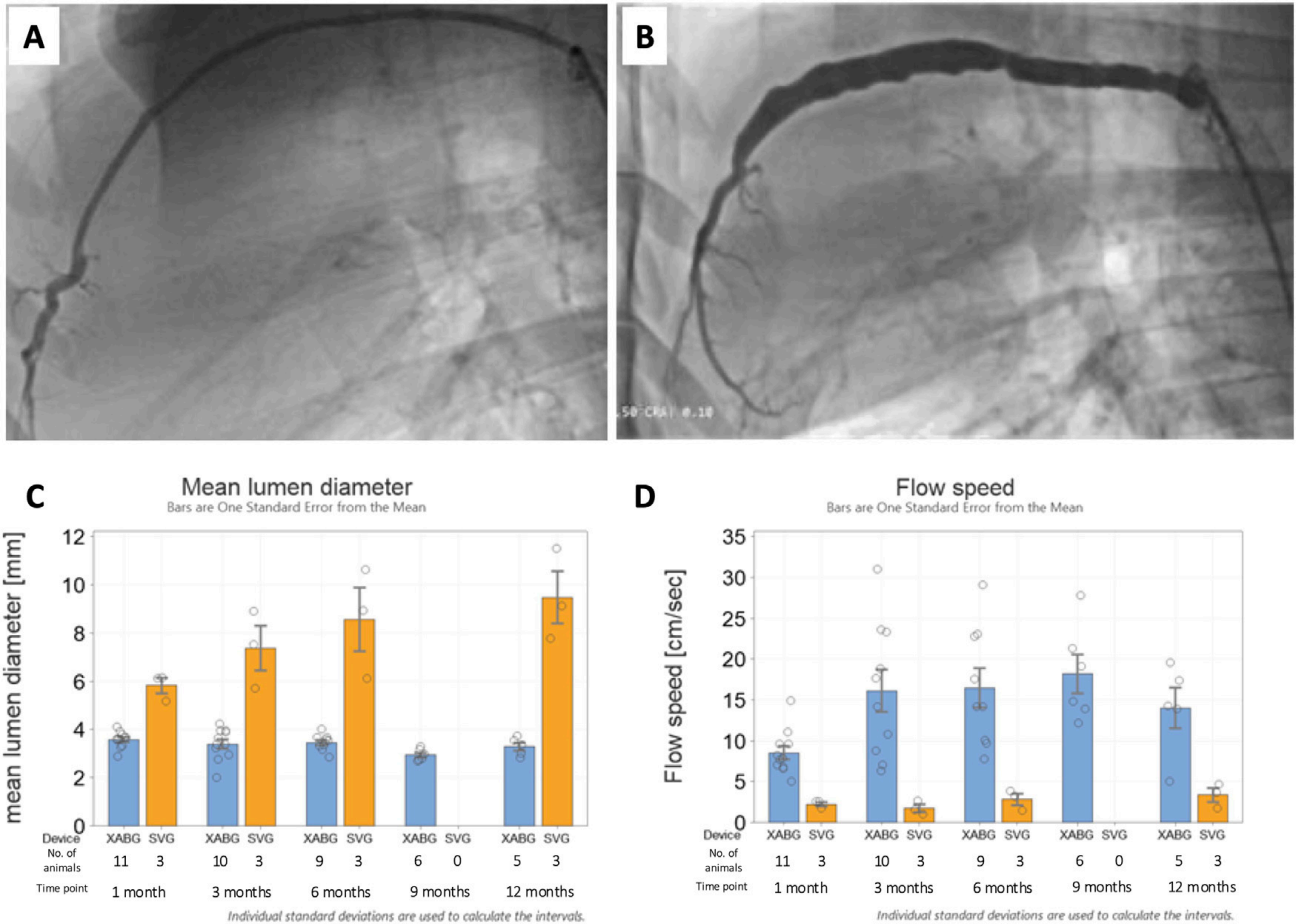
Representative 12-month angiography snapshots of an XABG **(A)** and SVG **(B)**. Serial mean lumen diameter for XABG vs. SVG **(C)**. Serial flow velocity for XABG vs. SVG **(D)**.

XABGs were generally characterized by uniform lumen profiles and stable mean diameters over time, while SVG controls had a more irregular profile with progressive dilatation (Figure 2). XABG flow velocity increased between 1 month and 3 months and remained stable with time, while flow velocity for SVG remained low through the study follow-up period. Note that detailed angiographic analysis has been reported separately (Ono et al., 2023; Poon et al., 2023).

### MicroCT morphometric analysis

**FIGURE 3:**
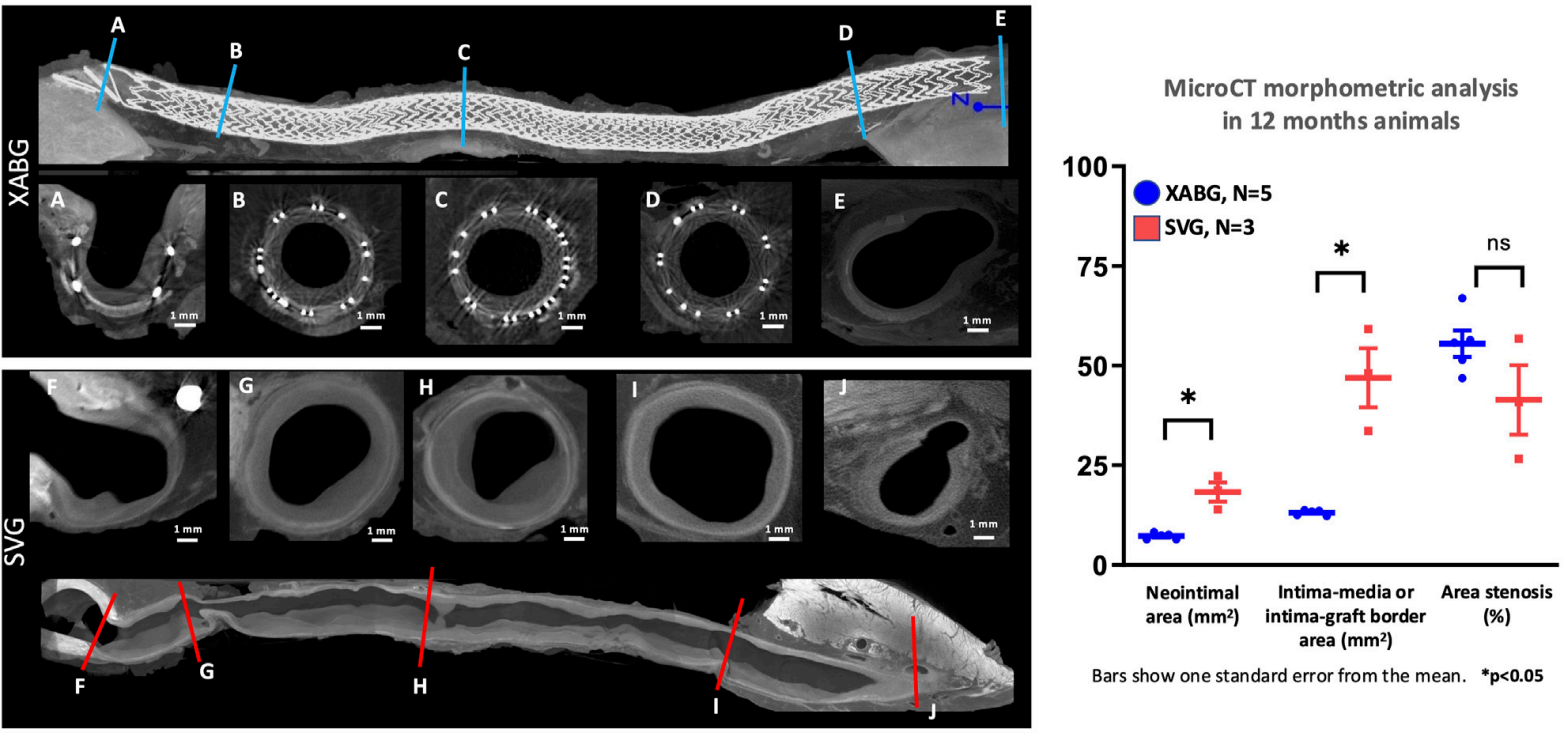
MicroCT cross-sectional images of XABG and SVG. Longitudinal microCT images of XABG (top) and SVG (bottom). Blue and red lines represent the sites of corresponding cross-sectional images of XABG **(A-E)** and SVG **(F-J)**, respectively. XABG shows consistently uniform vessel size from proximal to distal segments, while SVG shows enlargement and vessel size mismatch between graft middle **(B-D)** for XABG and **(G-I)** for SVG). The distal anastomosis cross-section **(E,J)** has an 8-like shape showing the XABG/vein on the left and the native coronary on the right. Note that the neointimal area (left graph) was greater in SVG than XABG; however, percent area stenosis was comparable between the grafts.

Explanted XABGs and SVGs underwent microCT image acquisition, confirming angiographic findings with a generally uniform lumen for XABGs and a more irregular and dilated luminal profile for SVGs (Figure 3). The graph in Figure 3 and Supplementary Table S3 shows the average result of morphometric analysis of the whole graft length. The intima-graft or intima-media border area, which represents a cross-sectional area of the vessels, was much greater in SVG than in XABG. More extensive neointima formation (i.e., neointimal area) can be observed in SVG than XABG [mean ± standard error (SE); SVG: 18.32 ± 2.41 mm^2^ vs. XABG: 7.26 ± 0.36 mm^2^, p = 0.0253], which is more prominent in the distal segment of the SVG group due to thrombus formation (Supplementary Table S4). Percent area stenosis was comparable between the two groups (41.42% ± 6.15% vs 55.51% ± 4.76%, p = 0.3).

### Gross findings

Grossly, all XABG devices demonstrated epicardial adhesions and coverage by epicardial fat and fibrous tissue. The grafts were fully expanded and remained uniformly circular without evidence of graft kinking. One XABG showed ectasia, while another showed the presence of an aneurysm in the middle of the graft.

### Microscopic findings

**FIGURE 4:**
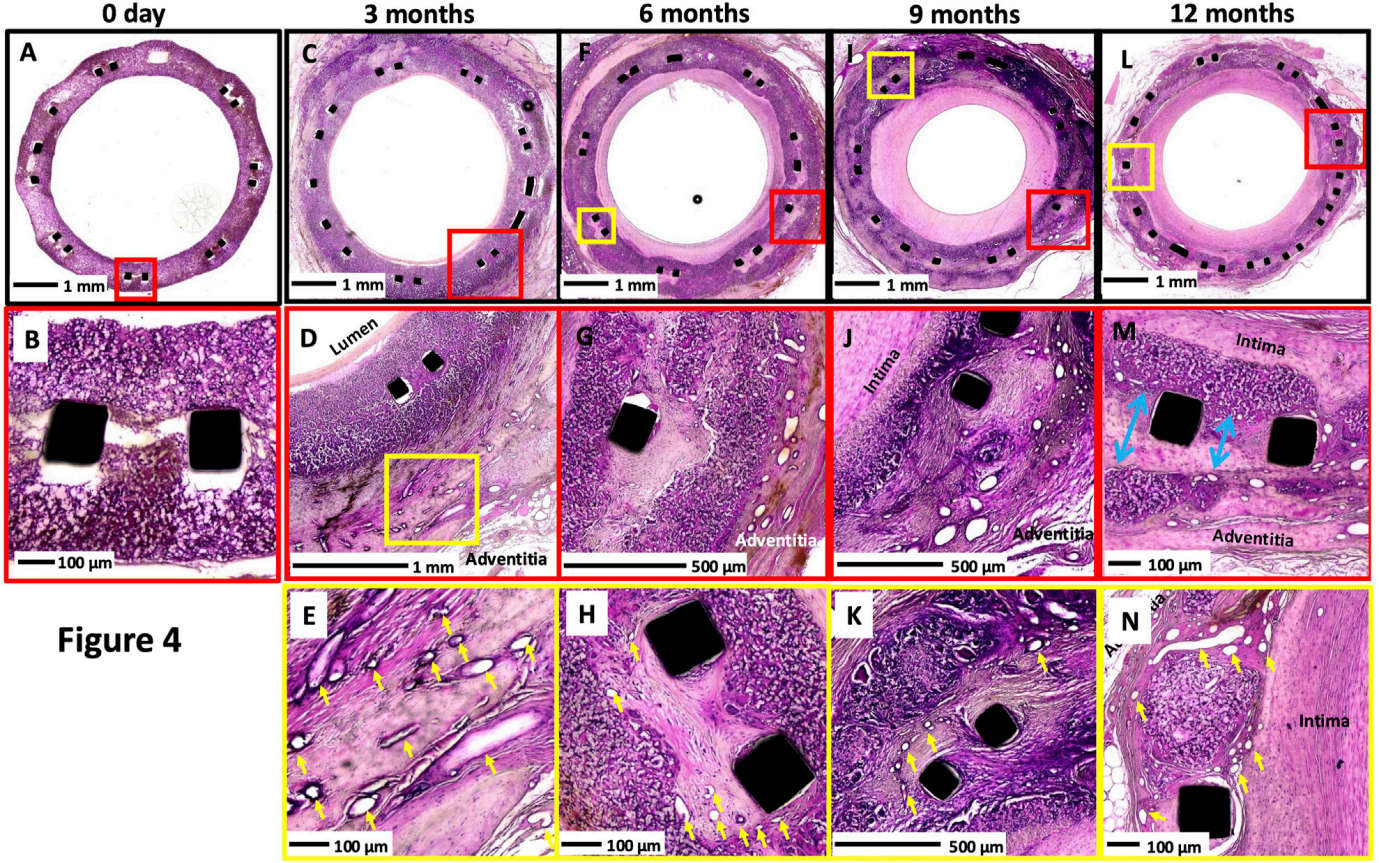
Representative histologic images of XABG at varying time points. Histologic images at low and high power of XABG sections at 0 days, **(A-B)**, 3 months **(C-E)**, 6 months **(F-H)**, 9 months (I to K), and 12 months **(L-N)**. Overall, neointimal growth increased over time and plateaued or regressed minimally at 12 months. Angiogenesis (yellow arrows) was observed as early as 3 months and was most prominent on the adventitial surface, with extension into the graft material and the neointima as well. Polymer degradation and collagen/proteoglycan matrix deposition were more frequently observed at later time points. Collagen deposition and polymer degradation were observed at the sites of separation of the polymer at the site of the metal implant (blue arrows in **(M)**). All sections were stained with H&E.

The polymer absorption and matrix deposition increased over time (Table 1). The area of resorption was replaced by smooth muscle cells surrounded by proteoglycan-rich collagen matrix with angiogenesis (Figure 4). At 12 months, greater degradation of the polymer with focal areas of polymer resorption was seen than at the earlier time points (Supplementary Figure S3).

**TABLE 1:** Summary of semi-quantitative scores of histologic responses of the XABG device.

	3 months	6 months	9 months	12 months	p-value
No. of animals	2	2	2	5	
Inflammation score	2.25 ± 0.46	3.13 ± 0.99	3.25 ± 0.46	2.65 ± 1.18	0.0975
Neovascularization	2.38 ± 0.52	2.5 ± 1.2	2.88 ± 0.64	2.9 ± 0.72	0.32
Fatty infiltrate	0 ± 0	0 ± 0	0 ± 0	0 ± 0	1
Necrosis	0 ± 0	0 ± 0	0 ± 0	0 ± 0	1
Matrix deposition	1.63 ± 0.74	1.5 ± 0.53	2.63 ± 0.92	2.5 ± 0.76	0.0037
Polymer absorption	1.25 ± 0.71	1.88 ± 1.13	2.13 ± 0.83	2.35 ± 0.75	0.0181
Calcification	0 ± 0	0.13 ± 0.35	0.5 ± 0.93	0.3 ± 0.73	0.52
Fibrin/thrombus	1.25 ± 1.49	1.25 ± 1.75	0.63 ± 1.41	0.15 ± 0.67	0.0293
Endothelialization	53.75 ± 50.41	51.25 ± 46.35	65.63 ± 47.62	96.75 ± 13.4	0.0165

Values are shown as mean ± standard error.

The inflammation increased over time up to 9 months and decreased thereafter; the score was not significant but trending (p = 0.0975) (Table 1). The inflammatory cells consisted predominantly of macrophages and foreign body giant cells and were almost always accompanied by angiogenesis. This is consistent with CD68 macrophage stains performed in earlier studies on the same polymer (Cramer et al., 2022). Note that macrophage IHC stains of the polymer were not performed in the current study due to plastic embedding limitations. Throughout the study, there were no indications of local or systemic adverse tissue response to the XABG conduit.

**FIGURE 5:**
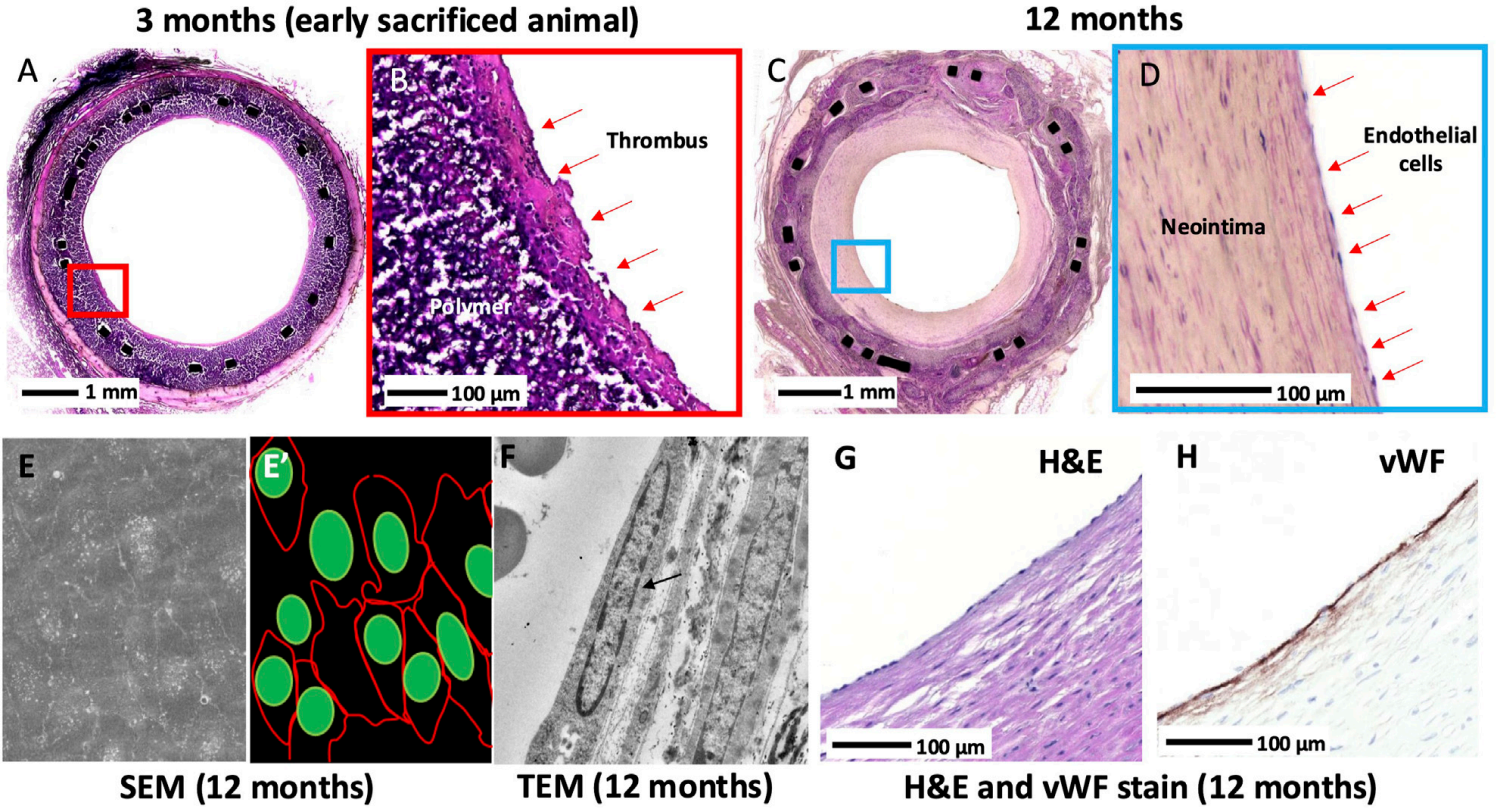
Histologic images of the luminal surface at 3 months and 12 months. Middle graft H&E-stained section from an early sacrificed animal at 3 months at low and high power **(A-B)**. The surface of the lumen is covered with a thrombus, and the endothelium is absent. Middle graft H&E-stained section from a 12-month animal at low and high power **(C-D)**. Neointimal growth and complete endothelialization are observed. Middle-graft SEM showed confluent endothelial cell lining with tight cell-to-cell junctions **(E)** and artistic illustration of cell junction (red) and cell nuclei (green) **(E’)**. TEM showed an endothelial cell nucleus (black arrow) lining the lumen with the absence of a thrombus **(F)**. A high-power H&E-stained image of the surface of the neointima taken from the middle graft showing surface endothelial lining of the luminal surface **(G)** and corresponding section **(H)** showed vWF stain-positive cells.

Endothelialization of the XABG surface was incomplete in early (up to 6 months) sacrificed animals with luminal thrombus, while in 12-month animals, almost complete endothelialization was observed (Table 1; Figure 5). SEM (N = 5 at 12 months) confirmed near confluent coverage with endothelial-like cells with focal thrombus, observed only in one of five grafts, and TEM (N = 1 at 12 months) showed endothelial cells on the luminal surface of the XABGs (Figure 5) with the presence of endothelial junctions. Paraffin-embedded sections of the carefully removed neointimal tissue from XABG (N = 2 at 12 months) showed positive vWF staining consistent with endothelial cell coverage (Figure 5). The neointima was rich in aSMA-positive cells consistent with a smooth muscle cell phenotype (data not shown).

### Histomorphometrical analysis

**FIGURE 6:**
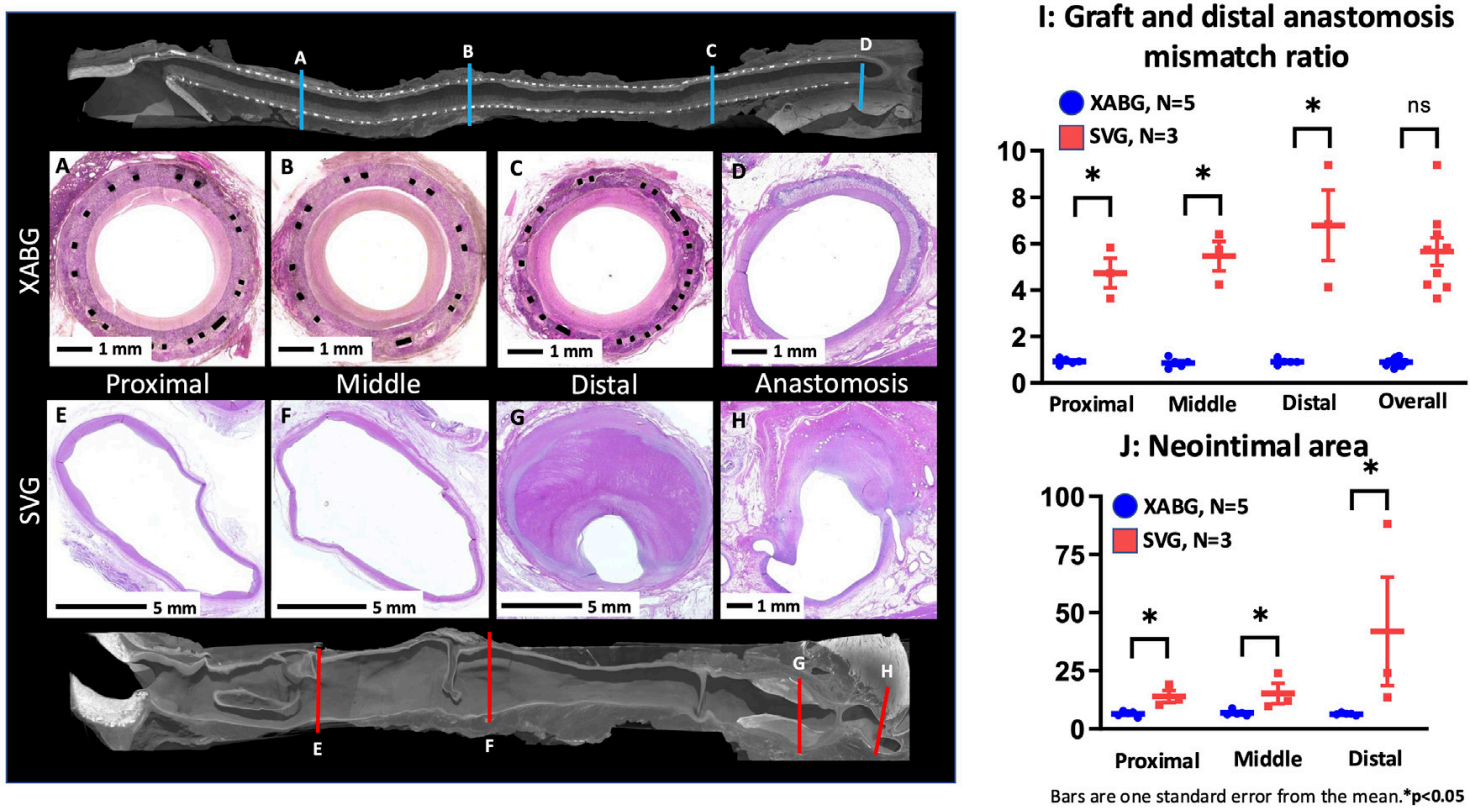
Representative microCT and histologic images of graft sections and distal anastomosis from the XABG and SVG. Note that the cross-sectional size of the XABG does not show the enlargement of the graft **(A-D)**, while SVG grafts are remarkably dilated (note the scale bars are 1 mm for XABGs and 5 mm for SVG graft sections), and the distal segment of SVG is filled with luminal thrombus **(E-H)**. The graft/distal anastomosis mismatch ratio **(I)** demonstrates that SVG segments show greater vessel enlargement than XABG segments. Neointimal area of XABG/SVGs in various regions **(J)**. SVGs demonstrated greater neointimal area than XABGs in all three locations.

Histomorphometry was performed in the animals with XABG (N = 5, 15 sections) and SVG (N = 3, 9 sections) (Supplementary Table S5). Percent area stenosis of both grafts was similar (mean ± SE; XABG 53.7% ± 5.1% vs SVG 41.4% ± 6.6%, p = 0.14); however, SVG showed greater vessel cross-sectional area than XABG (intima-media border area in SVG vs intima-graft border area in XABG; 60.8 ± 5.8 mm^2^ vs. 12.6 ± 4.5 mm^2^, p < 0.0001). The magnitude of neointima formation (i.e., neointimal area) was greater in SVG than XABG (23.7 ± 5.0 mm^2^ vs. 6.6 ± 3.9 mm^2^, p < 0.0001), especially in the distal segments of SVG due to luminal thrombus formation (Figures 6A–H, J; Supplementary Table S6). When comparing vessel size of graft sections from the proximal, mid, distal, and distal anastomosis, SVG was significantly dilated compared to XABG (Figure 6I). Therefore, the percent area stenosis of the two groups appears similar, although there is more neointimal tissue formation on the SVGs than on XABGs (Supplementary Tables S5, S6). These histomorphometry results are consistent with the results of microCT morphometric analysis.

## Discussion

The main findings of this study are as follows: (1) five of the seven XABG sheep that were kept beyond 6 months survived until 12 months with a patent graft; (2) angiographic, microCT, and histologic analysis of XABGs demonstrated uniform and a less ectatic profile with time than SVG controls; (3) the polymer absorption and matrix deposition increased over time without extensive inflammation or narrowing; (4) almost complete endothelialization of the luminal surface of XABG (SEM, TEM, and immunohistochemical analysis) was observed at 12 months. Because the XABG used in the study was 15 cm long, it is unlikely that mid-graft endothelialization was achieved through transanastomotic ingrowth (Zilla et al., 2007). Therefore, it seems reasonable that transmural ingrowth via angiogenesis from the abluminal surface of the graft was an important source of endothelialization, possibly in combination with fallout endothelialization from circulating cells (Sánchez et al., 2018), suggesting that the XABG is a viable alternative for bypass graft surgery. Notably, while endothelialization suggests advanced healing of the luminal surface, absorption of the polymer is not complete by 12 months. This is a consequence of the deliberate choice for a gradually absorbing polymer composition to ensure adequate mechanical support while the tissue gradually builds strength.

### Limitations of the engineered SDVG large animal model

Although prosthetic vascular conduits were introduced almost 70 years ago, no small-diameter (<6 mm) synthetic conduit has been approved for use as a CABG. Biological materials (e.g., bovine mammary arteries) were also used for SDVG; however, no studies showed successful results (Alexander et al., 2005). Although ePTFE has been successfully surgically implanted in patients with peripheral vascular disease and for hemodialysis access (Tzchori et al., 2018), rapid failure of ePTFE has been reported in CABG surgery (Chard et al., 1987). Importantly, ePTFE grafts are known to never fully endothelialize beyond ∼2 cm of the anastomotic site (Chard et al., 1987). To the best of our knowledge, this study is the first to evaluate a 4 mm SDVG, 15 cm in length, in an ovine model with a 12-month follow-up. Even with less challenging arterial bypass models (e.g., carotid interposition, femoral-femoral, or iliac-femoral) and relatively short graft lengths (typically 5–7 cm), patency of small-diameter synthetic grafts (≤4 mm) has reported 0%–25% patency in studies extending beyond 1 month follow-up (Fang et al., 2021). Our study, on the other hand, achieved 73% patency at 6 months, with near-complete endothelialization at 1 year, implanted in a low-flow environment of CABG.

### The mismatch between graft and distal anastomosis plays a role in graft patency

The vein-graft-to-native-artery anastomosis mismatch in both diameter and compliance are known contributors to SVG failure (Ghista and Kabinejadian, 2013). Various approaches to refining the patency rate of SVG have been conducted. Only external support devices for SVGs have gained momentum in clinical utility to date (Taggart et al., 2015). The experimental work by Taggart et al. demonstrated that by using an external stent to prevent dilation of SVGs, there is a significant reduction in intimal hyperplasia by attenuating hemodynamic disturbances caused by irregular lumen geometry (Taggart et al., 2015). However, the VEST trial failed to show the superiority of the device in the primary endpoint, which was the neointimal hyperplasia area as assessed by intravascular ultrasound at 12 months (Goldstein et al., 2022). XABG showed mild neointimal growth with surface endothelialization and less diameter and compliance mismatch, which may result in better long-term outcomes (Zilla et al., 2007; Taggart et al., 2015).

### Graft luminal surface thrombogenicity and endothelialization

Thrombosis remains a major cause of SDVG failure. Various types of modifications have been performed, and most have embraced those possessing antithrombotic properties, such as agents (e.g., heparin, nitric oxide, tissue-type plasminogen activator, and thrombomodulin) or recellularization by *in vitro* repopulation of a scaffold with endothelial cells (Fang et al., 2021; Tatterton et al., 2012). However, studies with high clinical relevance (use of large animal models) are still limited in number. The fundamental reason for thrombosis is the failure of the graft lumen to heal with a confluent endothelium. Although ePTFE grafts never fully endothelialize beyond ∼2 cm of the anastomotic site (Zilla et al., 2007), there are reports of small-diameter ePTFE grafts implanted in humans following pre-seeding with endothelial cells prior to implantation that can achieve chronic patency when used as CABG even as late as 27.7 months (Laube et al., 2000). Thus, if it were possible to line the lumen of a synthetic graft with endothelium, the thrombosis problem could be avoided. ePTFE (and other synthetic) graft architecture inhibits transmural endothelial cell migration, which is needed for the natural development of a confluent endothelium (Zilla et al., 2007; Bezuidenhout et al., 2002). Transmural microvessel connectivity within the graft, from the abluminal surface to the lumen, is essential to supply enough endothelial cells for a dense, confluent endothelium that prevents thrombosis (Zilla et al., 2007). Endothelial progenitor cell (EPC) recruitment is another possibility that has been shown to work with CD 34 antibody deposition on stents with limited neointimal growth at 9 months in humans (Aoki et al., 2005).

In general, the potential of fallout endothelialization in humans is considered limited compared to animal models (Zilla et al., 2007; Sánchez et al., 2018). The current sheep study does not separate fallout endothelialization from transmural ingrowth. Studies that separate both mechanisms by preventing abluminal ingrowth have demonstrated that fallout endothelialization is possible in various animal models (Talacua et al., 2015; Smith et al., 2020; Shi et al., 1994), although Pennel et al. demonstrated that endothelialization was far less when an abluminal wrap was used to prevent transmural ingrowth in a porous graft in a rat loop model (Pennel et al., 2018).

### Study limitations

Our research has some limitations. Not all animals survived for 1 year. Repeated angiography and intravascular OCT imaging may have contributed to early failures, which could have been avoided if intravascular imaging had not been part of the experimental setup. Our study was carried out in a healthy ovine model and did not include aged and diseased states, which exist in patients who require CABG. In the future, safety and efficacy will have to be demonstrated in a heterogeneous and diseased patient population. However, to our knowledge, the ovine CABG model has never been reported to survive more than 1 year. XABG was almost completely endothelialized and had less graft/coronary anastomotic mismatch (diameter and compliance) than the control SVGs; however, the 12-month cumulative patency rate was better with SVG than with XABG.

## Conclusion

The present study reports the patency of the Xeltis restorative bypass graft at 1 year. For the first time, we show mid-graft endothelialization with limited intimal thickening, bioresorption of the polymer, and replacement of the polymer by the animal’s own tissue in a challenging setting using the 15-cm-long and 4-mm-wide synthetic polymer conduit in the ovine CABG model. This study supports the safety of the Xeltis restorative bypass graft. Based on these data, approval was obtained for clinical investigation, which is currently ongoing in multiple centers in Europe (NCT04545112).

## Data Availability

The raw data supporting the conclusions of this article will be made available by the authors, without undue reservation.
