# Supplementary figures and images for: Histological assessment of a novel restorative coronary artery bypass graft in a chronic ovine model
Source: Front Bioeng Biotechnol. 2025 Feb 10;13:1488794. doi: 10.3389/fbioe.2025.1488794 (PMC11847836; doi:10.3389/fbioe.2025.1488794)

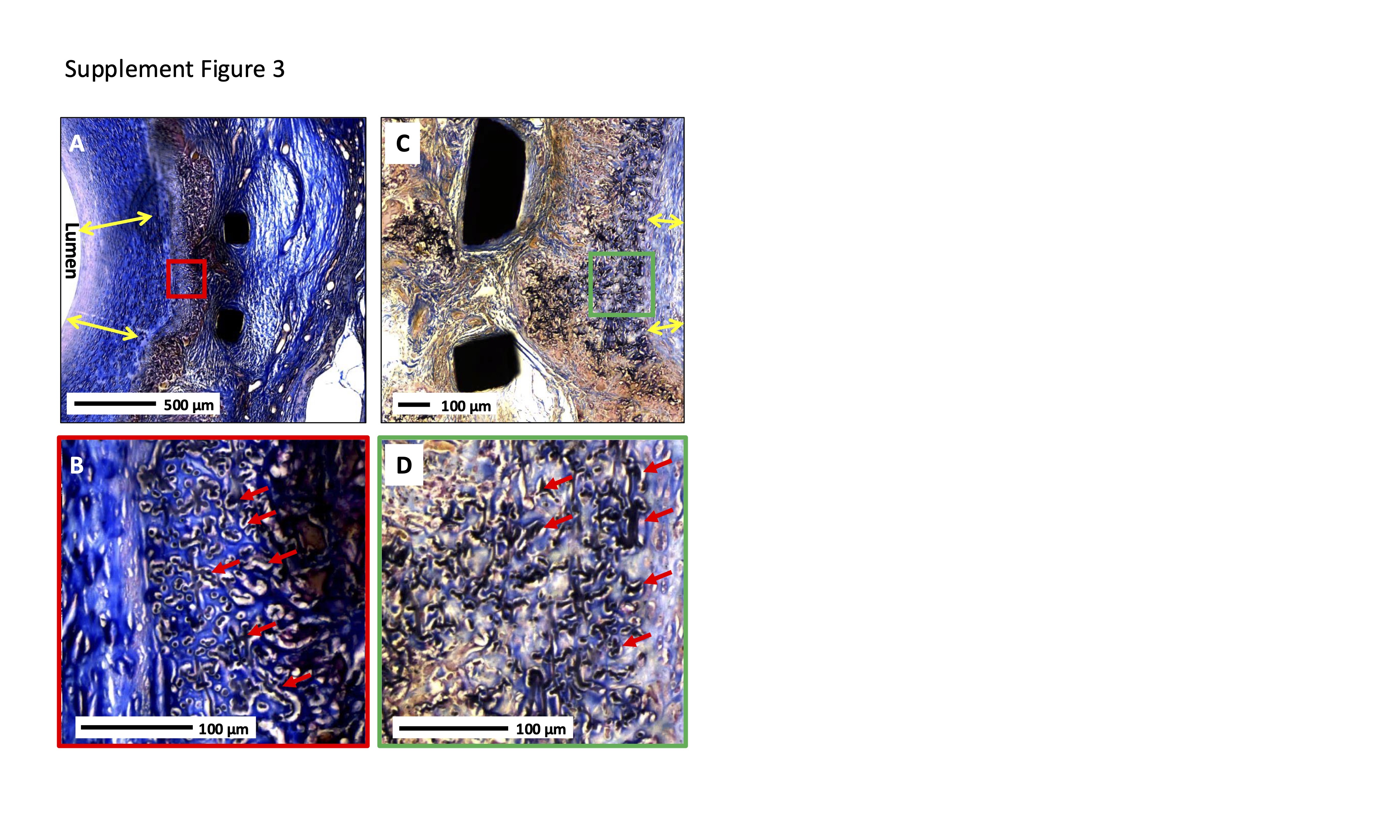

Supplement: Supplementary file 1 [file Image3.jpeg]

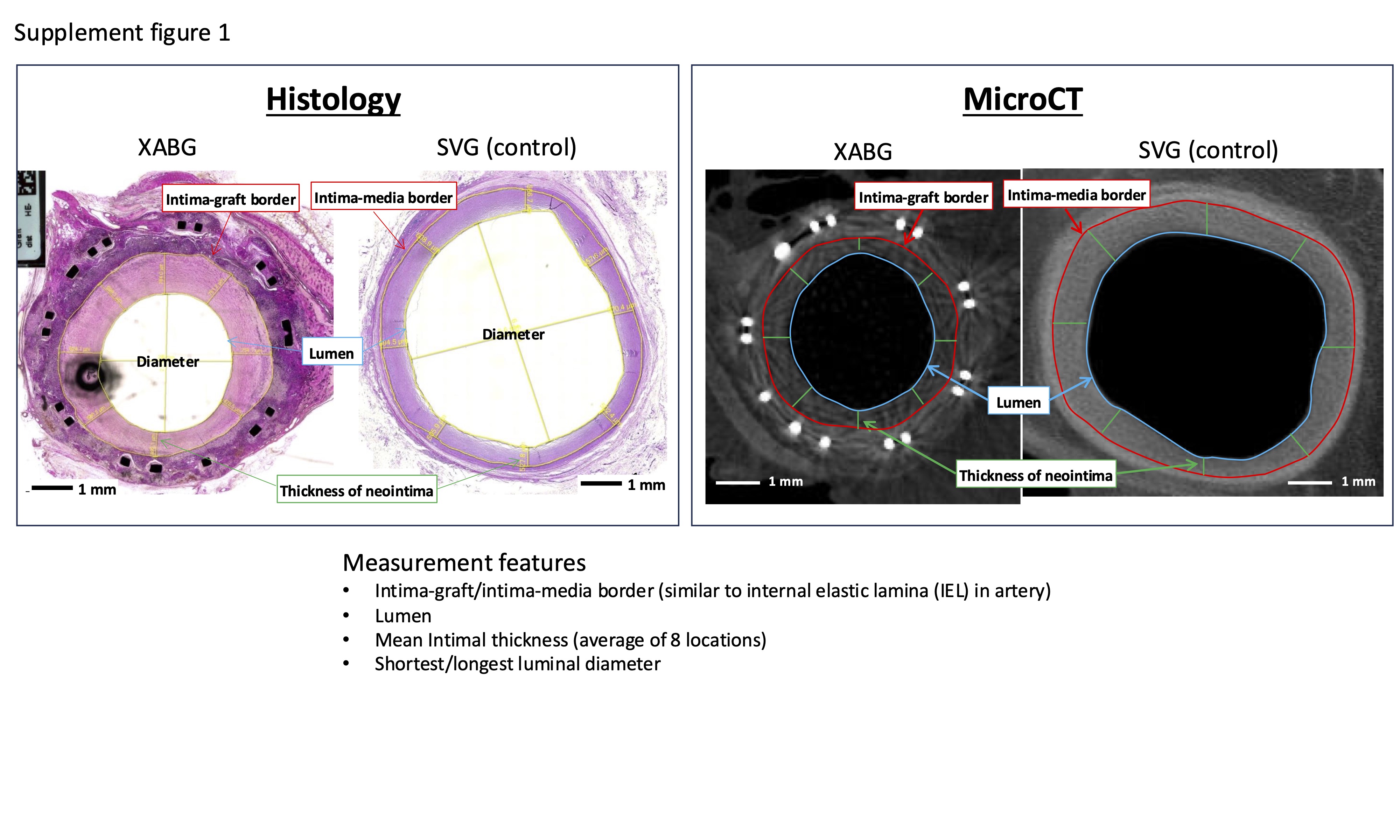

Supplement: Supplementary file 3 [file Image1.jpeg]

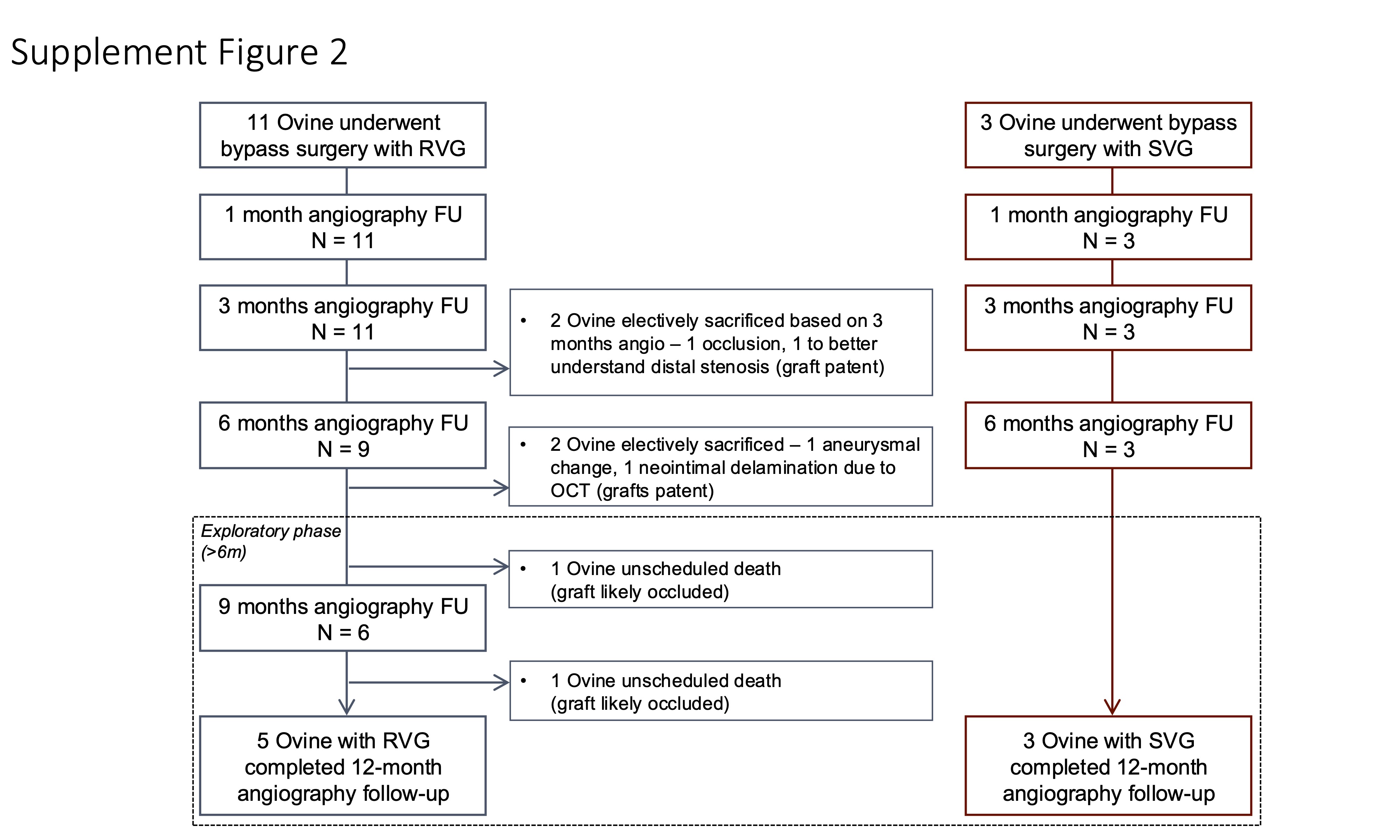

Supplement: Supplementary file 4 [file Image2.jpeg]
