# Supplementary material for: Histological assessment of a novel restorative coronary artery bypass graft in a chronic ovine model
Source: Front Bioeng Biotechnol. 2025 Feb 10;13:1488794. doi: 10.3389/fbioe.2025.1488794 (PMC11847836; doi:10.3389/fbioe.2025.1488794)
Supplement: Supplementary file 2 [file Table1.docx]

# Supplement Materials

## Supplement Methods

### MicroCT image acquisition and reconstruction

MicroCT image aquisition was performed using a Nikon X-Tek XT H 225ST Micro-CT system with a Perkin Elmer PE1621 EHS 2000x2000 X-ray detector panel (Nikon metrology, Brighton, MI, USA). Scanned images were exported as floating-point VGI volumes and rendered in VGSTUDIO MAX 3.0 (Volume Graphics GmbH, Heidelberg, Germany).

### Immunohistochemical staining

In selected XABG sections, immunohistochemical staining was performed to evaluate the surface endothelial lining. The inner intima of the middle segment of the XABG was carefully removed, embedded in paraffin, and stained with von Willebrand factor (vWF) antibody (A0082, dilution 1:1600, 20 min, Dako, Carpinteria, California, USA) and aSMA (760–2833, Ventana Medical Systems, Inc, Arizona, USA).

### Transmission electron microscopy (TEM) and scanning electron microscopy (SEM)

In selected cases, TEM and SEM analysis were performed for the evaluation of the luminal surface endothelial lining. Specimens were dehydrated in graded series of alcohol, critically point dried, and sputter-coated with gold. Digital images were acquired using a Hitachi Model S-3400N or SU3500 (Hitachi High-Technologies Science America, Inc., Northridge, California, USA). Low power images (x15 magnification) of the entire luminal surface of the XABG device were taken to assess the endothelialization. Stitched low-power montage images of the entire luminal surface were then assembled into a single image using proprietary software. Higher power images of regions of interest were also taken at incremental magnifications (50x, 200x, and 600x). Endothelial cells were identified as spindle or polygonal-shaped monolayer sheets in close apposition, a distinguishing feature from other cell types in en face preparations. For TEM, the selected XABG samples were taken from formalin preserved tissue and fixed with 2.5% glutaraldehyde in 0.1M sodium phosphate buffer at 4 degrees and then 1% osmium tetroxide was used for post-fixation. The samples were dehydrated by graded alcohol and embedded in EPON Resin. Ultrathin sections were cut at 100nm using a Leica Model EMUC6 ultramicrotome (Leica Microsystems, Inc., Buffalo Grove, IL) and stained with 4% uranyl acetate and Reynolds lead citrate. The specimens were assessed with the TEM (Hitachi H-7650, Hitachi Science System Ltd., Japan).

### Semi-quantitative scoring and histomorphometry analysis

Ordinal data was collected for each conduit from the proximal and distal anastomoses and mid-sections of the XABG device with the severest and mildest change. The conduits were each semi-quantitatively graded with light microscopy with regard to specific pathologic features, which include architectural changes (matrix absorption and proteoglycan/collagen formation as an indicator of healing in the region of absorbed scaffold) as well as calcification, inflammation, fatty infiltrate, necrosis, and thrombi/vegetations (Supplement Tables 1 and 2). Score 0 = none, 1 = minimal, 2 = mild, 3 = moderate, 4 = severe. The semi-quantitative scoring did not apply to the control vein graft cases because there was no polymer conduit.

For microCT morphometric analysis, graft cross-section images were captured every 10 mm from the proximal to distal anastomosis. The luminal and intima-graft border areas were measured, and neointimal area and percent area stenosis were subsequently calculated (Supplement Figure 1). In histomorphometry analysis, digitized histology slides were utilized to measure luminal and intima-media border areas. Neointimal area and percent area stenosis were then calculated accordingly (Supplement Figure 1). The neointimal area was determined by subtracting the intima-graft/intima-media border area from the lumen area. Percent area stenosis was derived by dividing the neointimal area by the intima-graft or intima-media border area. Ostial sections were excluded from the analysis due to their longitudinal cut and inability to measure these features.

## Supplement Tables

- **Supplement Table 1: Definition of semi-quantitative scores**

| **Response** | **Score** | | | | | |
| --- | --- | --- | --- | --- | --- | --- |
|  | **0** | **1** | **2** | **3** | **4** | **5** |
| **Neovascularization** | 0 | Minimal capillary proliferation, focal, 1-3 buds | Groups of 4-7 capillaries with supporting fibroblastic structures | Broad band of capillaries with supporting structures | Extensive band of capillaries with supporting fibroblastic structures | NA |
| **Fatty Infiltrate** | 0 | Minimal amount of fat associated with fibrosis | Several layers of fat and fibrosis | Elongated broad accumulation of fat cells about the implant site | Extensive fat completely surrounding the implant | NA |
| **Necrosis** | 0 | Minimal | Mild | Moderate | Severe | NA |
| **Matrix absorption score** | No absorption of matrix | < 10% conduit area shows matrix absorption | 10-<25% conduit area shows matrix absorption | 25-<50% conduit area shows matrix absorption | 50-<75% conduit area shows matrix absorption | >75% conduit area shows matrix absorption |
| **Conduit matrix deposition (Fibrosis)** | No collagen replacement | Collagen replacement <10% conduit area | Collagen replacement 10-<25% conduit area | Collagen replacement 25%-<50% conduit area | Collagen replacement 50%-<75% conduit area | Collagen replacement >75% conduit area |
| **Surface Fibrin/Thrombus Score** | No surface thrombus deposition | Minimal fibrin-platelet/thrombus deposition involving <10% of the cross-sectional luminal area stenosis | Mild fibrin-platelet/thrombus deposition involving 10% to 25% of the cross-sectional luminal area stenosis | Moderate fibrin-platelet/thrombus deposition involving >25% to 50% of the cross-sectional luminal area stenosis | Heavy fibrin-platelet /thrombus deposition involving >50% of the cross-sectional luminal area stenosis | NA |

- **Supplement Table 2: Definition of semi-quantitative scores for inflammation**

|  | **Focal** | **Multifocal** | **Diffuse** |
| --- | --- | --- | --- |
| **Absent** | 0 | 0 | 0 |
| **Rare inflammatory cells infiltration** | 0 | 1 | 2 |
| **Mild infiltrate and is not the predominant component** | 1 | 2 | 3 |
| **Infiltrates up to ½ of the conduit** | 2 | 3 | 4 |
| **Infiltrates > ½ of the conduit** | 3 | 4 | 4 |

- **Supplement Table 3: Summary data of microCT morphometric measures of SVG and XABG in animals followed for one year**

| Group | XABG | SVG | p-value |
| --- | --- | --- | --- |
| Number of animals | 5 | 3 |  |
| Number of sections | 61 | 45 |  |
| Intima-graft/intima-media border area (mm^2^) | 13.1± 3.3 | 47.0 ± 4.3 | 0.0369 |
| Neointimal area (mm^2^) | 7.3 ± 1.1 | 18.3 ± 1.4 | 0.0369 |
| Area Stenosis (%) | 55.5 ± 7.5 | 41.4 ± 15.1 | 0.3 |

Values are shown as mean ± standard error.

**Supplement Table 4: Each location results of microCT morphometric measures of SVG and XABG in animals followed for one year**

| Location | Proximal | | Middle | | Distal | |
| --- | --- | --- | --- | --- | --- | --- |
| Group | XABG | SVG | XABG | SVG | XABG | SVG |
| Number of sections | 19 | 11 | 23 | 15 | 19 | 11 |
| Intima-graft/intima-media border area (mm^2^) | 13.2 ± 0.1 | 37 ± 3.9 | 13.4 ± 0.2 | 42.7 ± 4 | 12.9 ± 0.2 | 62.9 ± 10.1 |
| Neointimal area (mm^2^) | 7.2 ± 0.2 | 13.6 ± 1.6 | 7.8 ± 0.4 | 16.2 ± 1.1 | 6.9 ± 0.2 | 26.2 ± 8.2 |
| Area Stenosis (%) | 54.6 ± 1.7 | 40.6 ± 6 | 58.4 ± 3.2 | 42.8 ± 4.8 | 53.8 ± 2 | 40.3 ± 7.4 |

- **Supplement Table 5: Summary data of histomorphometric measures of SVG and XABG in animals followed for one year**

| Group | XABG | SVG | p-value |
| --- | --- | --- | --- |
| Number of animals | 5 | 3 |  |
| Number of sections | 15 | 9 |  |
| Intima-graft/intima-media border area (mm^2^) | 12.6 ± 4.5 | 60.8 ± 5.8 | <0.0001 |
| Lumen area (mm^2^) | 6.0 ± 4.5 | 37.1 ± 5.8 | <0.0001 |
| Neointimal area (mm^2^) | 6.6 ± 3.9 | 23.7 ± 5.0 | <0.0001 |
| Area stenosis (%) | 53.7 ± 5.1 | 41.4 ± 6.6 | 0.14 |
| Min diameter (mm) | 2.6 ± 0.3 | 4.5 ± 0.4 | 0.0185 |
| Max diameter (mm) | 2.8 ± 0.5 | 7.4 ± 0.6 | <0.0001 |
| Mean diameter (mm) | 2.7 ± 0.4 | 5.9 ± 0.5 | <0.0001 |
| Mean neointimal thickness (mm) | 0.6 ± 0.2 | 1.2 ± 0.2 | 0.28 |

Values are shown as mean ± standard error.

**Supplement Table 6: Each location results of histomorphometric measures of SVG and XABG in animals followed for one year**

| Location | Proximal | | Middle | | Distal | |
| --- | --- | --- | --- | --- | --- | --- |
| Group | XABG | SVG | XABG | SVG | XABG | SVG |
| Number of sections | 5 | 3 | 5 | 3 | 5 | 3 |
| Intima-graft/intima-media border area (mm^2^) | 12.9 ± 0.2 | 51.8 ± 17 | 12 ± 1.2 | 59.1 ± 17.8 | 12.8 ± 0.5 | 71.6 ± 20 |
| Lumen area (mm^2^) | 6.4 ± 0.7 | 37.8 ± 19.2 | 5.2 ± 1.3 | 43.9 ± 21.5 | 6.4 ± 0.4 | 29.6 ± 14.4 |
| Neointimal area (mm^2^) | 6.5 ± 0.5 | 14 ± 2.6 | 6.9 ± 0.5 | 15.2 ± 4.4 | 6.4 ± 0.2 | 42 ± 23.3 |
| Area Stenosis (%) | 50.4 ± 4.5 | 36.1 ± 15.6 | 60.4 ± 9.2 | 35.1 ± 18.4 | 50.4 ± 1.9 | 53.1 ± 18 |
| Min diameter (mm) | 2.8 ± 0.1 | 4.7 ± 0.6 | 2.2 ± 0.5 | 4.7 ± 1.3 | 2.7 ± 0.1 | 4.1 ± 1.8 |
| Max diameter (mm) | 2.9 ± 0.1 | 7.8 ± 2.3 | 2.7 ± 0.3 | 8 ± 2.1 | 3 ± 0.1 | 6.4 ± 1.5 |
| Mean diameter (mm) | 2.8 ± 0.1 | 6.3 ± 1.3 | 2.4 ± 0.4 | 6.3 ± 1.7 | 2.8 ± 0.1 | 5.2 ± 1.6 |
| Mean neointimal thickness (mm) | 0.6 ± 0.1 | 0.8 ± 0.2 | 0.7 ± 0.1 | 0.9 ± 0.3 | 0.6 ± 0 | 2.1 ± 1 |

Values are shown as mean ± standard error. *4 sections per animal, 1 each from proximal, middle, distal, and distal anastomosis.

## Supplement Figure Legend:

**Supplement Figure 1:** Histology and microCT morphometric measurement methods.

**Supplement Figure 2:** Study flow chart. Modified and reproduced from Ono M, et al. ( JACC Basic Transl Sci 2023;8:19-34).

**Supplement Figure 3:** Representative images of the polymer degradation and collagen deposition. Low (A and C) and high power (B and D) images of XABG stained with trichrome elastic stain. Collagen deposition (blue) surrounding the graft material (black color, red arrows) with absorption and replacement. The neointimal tissue matrix is predominantly made up of collagen (blue color) (yellow double arrows point to the region of neointima) (B and D).
